# Masks Start to Drop: Suppressor of MAX2 1-Like Proteins Reveal Their Many Faces

**DOI:** 10.3389/fpls.2022.887232

**Published:** 2022-05-12

**Authors:** Arne Temmerman, Ambre Guillory, Sandrine Bonhomme, Sofie Goormachtig, Sylwia Struk

**Affiliations:** ^1^Department of Plant Biotechnology and Bioinformatics, Ghent University, Ghent, Belgium; ^2^VIB-Center for Plant Systems Biology, Ghent, Belgium; ^3^Université Paris-Saclay, INRAE, AgroParisTech, Institut Jean-Pierre Bourgin (IJPB), Versailles, France; ^4^LIPME, Université de Toulouse, INRAE, CNRS, Castanet-Tolosan, France

**Keywords:** SMXL, strigolactones, karrikins, phylogenetics, biomolecular condensates

## Abstract

Although the main players of the strigolactone (SL) signaling pathway have been characterized genetically, how they regulate plant development is still poorly understood. Of central importance are the SUPPRESSOR OF MAX2 1-LIKE (SMXL) proteins that belong to a family of eight members in *Arabidopsis thaliana*, of which one subclade is involved in SL signaling and another one in the pathway of the chemically related karrikins. Through proteasomal degradation of these SMXLs, triggered by either DWARF14 (D14) or KARRIKIN INSENSITIVE2 (KAI2), several physiological processes are controlled, such as, among others, shoot and root architecture, seed germination, and seedling photomorphogenesis. Yet another clade has been shown to be involved in vascular development, independently of the D14 and KAI2 actions and not relying on proteasomal degradation. Despite their role in several aspects of plant development, the exact molecular mechanisms by which SMXLs regulate them are not completely unraveled. To fill the major knowledge gap in understanding D14 and KAI2 signaling, SMXLs are intensively studied, making it challenging to combine all the insights into a coherent characterization of these important proteins. To this end, this review provides an in-depth exploration of the recent data regarding their physiological function, evolution, structure, and molecular mechanism. In addition, we propose a selection of future perspectives, focusing on the apparent localization of SMXLs in subnuclear speckles, as observed in transient expression assays, which we couple to recent advances in the field of biomolecular condensates and liquid–liquid phase separation.

## Introduction

### Strigolactones Signal Through D14 and MAX2

Plants continuously tailor their growth to a vast array of external and internal stimuli, which are integrated and translated into a developmental output by the interplay of several endogenous signaling molecules. Numerous aspects of plant development are modulated by one of the most recently characterized class of phytohormones, strigolactones (SLs; reviewed in Aquino et al., 2021). However, SLs had originally been discovered as rhizosphere signals that enable the interaction between the plant host and symbiotic organisms, both parasitic, i.e., root-parasitic plants from the Orobanchaceae family (reviewed in Bouwmeester et al., 2021), and mutualistic, i.e., arbuscular mycorrhizal fungi (reviewed in Lanfranco et al., 2018). Thus far, more than 30 different SLs have been identified in a multitude of plant species (Yoneyama et al., 2018; Xie et al., 2019). Initially, only compounds, now referred to as canonical SLs, consisting of a tricyclic ABC scaffold connected through an enol ether bridge to a butenolide D-ring, were considered as SLs. Based on the configuration of the stereocenter between the B- and C-rings, canonical SLs can be subdivided in strigol-like and orobanchol-like molecules (Wang and Bouwmeester, 2018). More recent discoveries revealed the existence of noncanonical SLs, in which the D-ring is attached to a chemical structure different from the canonical ABC scaffold (Yoneyama et al., 2018). All natural SLs contain a stereocenter at the 2′ position of the D-ring, which is set in an R configuration (Flematti et al., 2016). In contrast, the most extensively used SL analog, *rac*-GR24, is synthesized as a racemic mixture consisting of both the 2’R and 2’S enantiomers, each with a distinct functionality in plant growth. The current nomenclature of GR24 isomers refers to a stereotypic strigol-like (5-deoxystrigol; 5DS) or orobanchol-like (4-deoxyorobanchol; 4DO) compound, thus specifying the configuration of the ABC rings.

**Figure 1:**
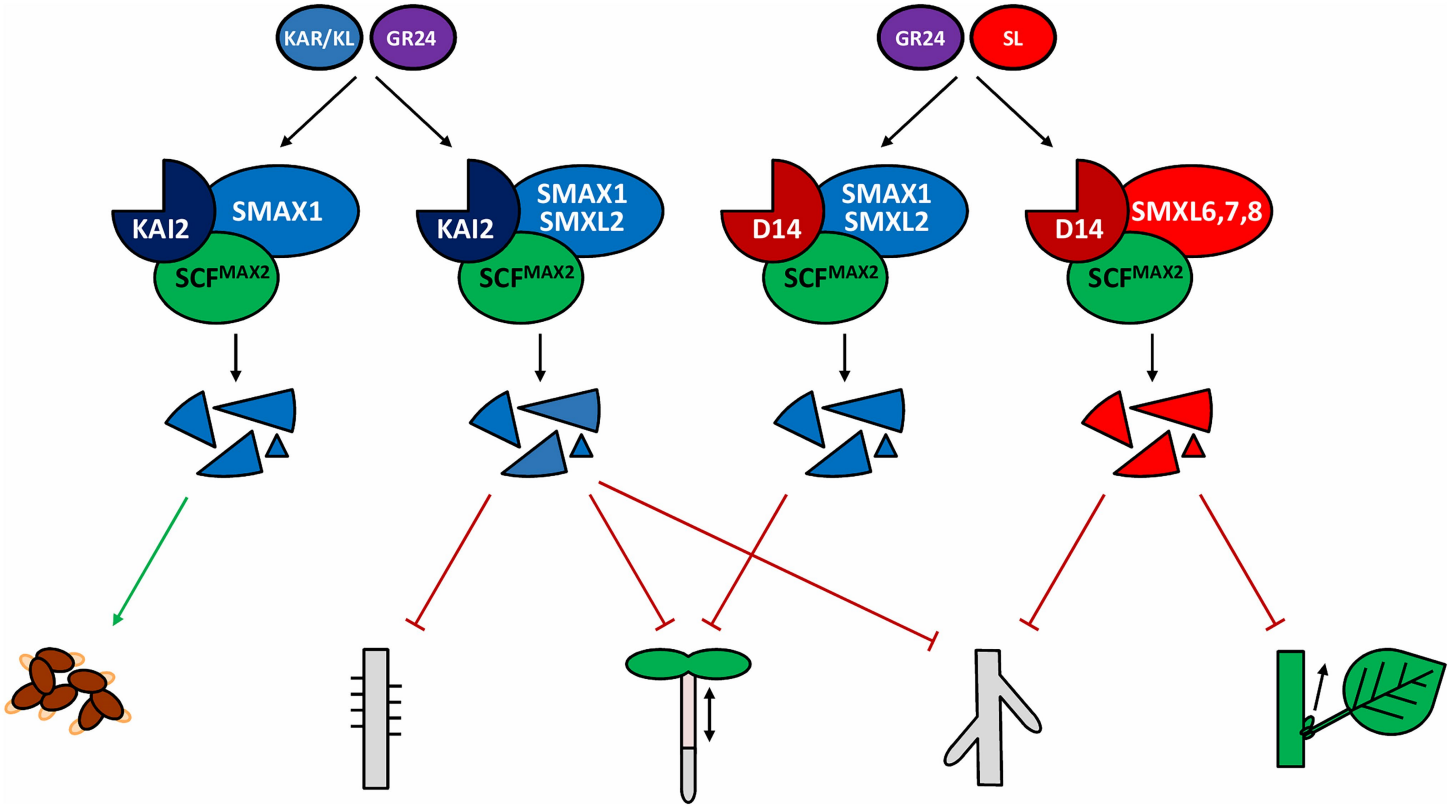
Core D14 and KAI2 signaling pathways. The signaling complexes formed after the perception of their respective ligands as well as a selection of phenotypes affected by the SMXL protein degradation are shown, induction of seed germination induction, inhibition of root hair development, hypocotyl elongation, lateral root formation, and shoot branching. A more extensive list of processes regulated by these pathways can be found in Table 1. GR24, *rac*-GR24; KL, KAI2 ligand; SL, strigolactone.

In angiosperms, SLs are perceived by the dual function receptor/enzyme DWARF14 (D14), a member of the α/β-fold hydrolase superfamily (Figure 1; Hamiaux et al., 2012; Zhao et al., 2013; de Saint Germain et al., 2016; Yao et al., 2016). A characterizing feature of α/β hydrolases is the presence of a conserved catalytic serine-histidine-aspartic acid (Ser-His-Asp) triad. Based on the crystal structures of the *Arabidopsis thaliana* and *Oryza sativa* (rice) D14 homologs, a mode of action had been suggested, in which D14 hydrolyzes the SL molecule, opening the D-ring and detaching it from the ABC scaffold that subsequently leaves the catalytic site. The open D-ring is covalently bound to the catalytic Ser residue and finally transferred to the catalytic His, through the formation of a ‘covalently linked intermediate molecule’ (CLIM; Nakamura et al., 2013; Zhao et al., 2013, 2015; Yao et al., 2016; Shabek et al., 2018). Although hydrolysis of SLs had initially been hypothesized as essential to convert D14 into an active state, later evidence resulted in the competing hypothesis that D14 becomes active upon binding of SL, whereas hydrolysis merely deactivates the bioactive molecule (Seto et al., 2019). To date, the precise function of SL hydrolysis and the nature and role of the covalent modifications of D14 remain open questions (Bürger and Chory, 2020).

After perception of its ligand, further signal transduction relies on the D14-mediated recruitment of the F-box protein MORE AXILLARY GROWTH 2 (MAX2; Figure 1; Stirnberg et al., 2007; Waters et al., 2012). As a part of an SKP-CULLIN-F-box (SCF) complex, MAX2 is responsible for the polyubiquitination of certain target proteins from the SUPPRESSOR OF MAX2 (SMAX)1-LIKE (SMXL) family, which are consequently degraded by the 26S proteasome, resulting in downstream signaling (Stirnberg et al., 2007; Jiang et al., 2013; Stanga et al., 2013; Zhou et al., 2013; Soundappan et al., 2015). Interaction between MAX2 and D14 involves a cycle of concerted conformational changes of both proteins, partly determining whether D14 continues to activate signaling or is degraded in a MAX2-dependent manner (Chevalier et al., 2014; Hu et al., 2017; Shabek et al., 2018). Crystal structure studies in rice revealed that this balance depends on DWARF3 (D3) and DWARF53 (D53), the rice homologs of MAX2 and SMXL6/7/8, respectively. Indeed, D3 switches between two functional conformations, characterized by different positions of its C-terminal α-helix (CTH) that can either be engaged with or dislodged from the remainder of the protein (Shabek et al., 2018). When D14 binds bioactive SLs, D3 with a dislodged CTH will interact with and hold D14 in an open and enzymatically inactive conformation, until D53, cooperatively bound by D14 and the D3-CTH, is recruited to the signaling complex (Shabek et al., 2018). This tripartite interaction will trigger the D14 enzymatic activity. Hence, D14 will hydrolyze SLs and switch to a closed conformation, in turn converting D3 to its CTH-engaged form. In this form, the signaling complex will ubiquitinate D53, resulting in its degradation and removal from the complex (Jiang et al., 2013; Zhou et al., 2013; Shabek et al., 2018). Finally, D14 itself is also polyubiquitinated and proteasomally degraded. The current knowledge on D14 ligand perception and hydrolysis, as well as the formation of the D14-MAX2-SMXL complex and activation of SL signaling, have been recently reviewed in detail (Bürger and Chory, 2020). Nevertheless, the exact stoichiometry of the process remains unclear: how many SL molecules can D14 hydrolyze before it gets degraded? How many SMXL proteins can be marked for degradation for each hydrolyzed SL molecule (Shabek et al., 2018)?

### The D14 Homolog KAI2 Induces a Parallel Signaling Pathway

MAX2 is important not only for SL signaling but also for the response to a class of exogenous compounds, karrikins (KARs), produced from burned plant material (Flematti et al., 2004; Nelson et al., 2009, 2011). This observation was followed by the discovery of another α/β hydrolase and D14 paralog, KARRIKIN INSENSITIVE 2 (KAI2) or HYPOSENSITIVE TO LIGHT (HTL), that acts as a KAR receptor (Sun and Ni, 2011; Waters et al., 2012). In a pathway similar to that of D14-MAX2, perception of KARs by KAI2 results in the recruitment of the SCF^MAX2^ complex and marking for proteasomal degradation of SMXL proteins (Figure 1; Nelson et al., 2011; Stanga et al., 2013, 2016; Khosla et al., 2020; Wang et al., 2020b). Despite the use of highly related components, D14- and KAI2 signaling regulate distinct, but overlapping sets of developmental outputs (see below; De Cuyper et al., 2017; Machin et al., 2020). As already established, both pathways also have distinct inputs; considering exogenous compounds, D14 is generally responsive to 2’R-configured SLs and the SL analogs GR24^5DS^ and GR24^4DO^, whereas KAI2 responds to KARs and the 2’S-configured GR24^ent-5DS^ (Scaffidi et al., 2014; Waters et al., 2015b; Flematti et al., 2016). In contrast to D14, KAI2 is found in all sequenced land plant genomes and in some charophyte algae, suggesting that KAI2-MAX2-dependent signaling is ancestral and that D14 probably evolved through duplication and neofunctionalization of KAI2 (Delaux et al., 2012; Bythell-Douglas et al., 2017). Interestingly, the ability to perceive SLs has been proposed to have arisen at least additionally twice in the evolution of land plants, because both the moss *Physcomitrium patens* and the parasitic plant species from the Orobanchaceae family possess KAI2-like SL-sensitive receptors (Conn et al., 2015; Xu et al., 2018; Lopez-Obando et al., 2021).

Despite its ubiquity in land plant species, ligand perception by KAI2 is much less understood than that of D14. Although the Ser-His-Asp triad of KAI2 was found necessary for signaling and KAI2 displays hydrolytic activity toward GR24^ent-5DS^, KARs are not susceptible to such hydrolysis (Scaffidi et al., 2012; Waters et al., 2015b; Yao et al., 2018). Additionally, the precise orientation in which KAR molecules bind in the catalytic pocket is inconsistent in crystal structures of different KAI2 homologs (Guo et al., 2013; Xu et al., 2016). As KARs also generally appeared unable to activate KAI2 in assays outside the plant cell, they have been suggested to require some unknown *in planta* metabolic steps to turn them into suitable KAI2 ligands (Guo et al., 2013; Nakamura et al., 2013; Waters et al., 2015a; Xu et al., 2016, 2018; Yao et al., 2018; Khosla et al., 2020; Wang et al., 2020b). Moreover, the currently reigning hypothesis states that both KARs and GR24^ent-5DS^ are merely substitutes for endogenous KAI2 ligands (KLs; Waters et al., 2012; Conn and Nelson, 2016). Despite many independent lines of evidence supporting their existence, KLs have not been detected yet, and their nature is still unknown (Nelson et al., 2011; Conn et al., 2015; Waters et al., 2015b; Sun et al., 2016). Similar to D14, KAI2 is also subjected to ligand-induced degradation, but its degradation has been shown to be independent of MAX2 and the 26S proteasome (Chevalier et al., 2014; Waters et al., 2015a; Hu et al., 2017; Yao et al., 2018). For both receptors, the role this degradation plays in signaling is still unclear.

### SMXL Proteins Regulate a Wide Variety of Physiological Processes

In *Arabidopsis*, the family of SMXLs consists of eight members, classified into four phylogenetic subclades, SMAX1/SMXL2, SMXL3, SMXL4/5, and SMXL6/7/8, which also largely correspond to their functions (Stanga et al., 2013; Moturu et al., 2018; Walker et al., 2019). SMXL6/7/8 are the target proteins first described as being ubiquitinated and degraded upon SL-activated D14-MAX2 signaling. This pathway regulates several physiological processes, including inhibition of shoot branching (Soundappan et al., 2015; Wang et al., 2015), cotyledon expansion (Soundappan et al., 2015) and lateral root outgrowth (Soundappan et al., 2015; Villaécija-Aguilar et al., 2019); increase of branch angle (Liang et al., 2016) and leaf and petiole length (Soundappan et al., 2015); promotion of stem elongation (Soundappan et al., 2015; Liang et al., 2016), leaf senescence (Bennett et al., 2016) and secondary growth (Liang et al., 2016); and protection against drought stress through stomatal closure (Yang et al., 2020a), thickening of the cuticle and production of anthocyanins (Figure 1; Table 1; Li et al., 2020). The role for SMXL6/7/8 in shoot branching or tillering in monocotylednous plants, has been studied in several additional species, including rice, wheat (*Triticum aestivum*), and pea (*Pisum sativum*), suggesting that this role for SL signaling is conserved at least across angiosperms (Jiang et al., 2013; Zhou et al., 2013; Liu et al., 2017; Kerr et al., 2021). In apple (*Malus domestica*) and woodland strawberry (*Fragaria vesca*), SMXL6/7/8 has been inferred to play a role in abiotic stress and flower development respectively, although not yet confirmed by functional characterization (Li et al., 2018; Wu et al., 2019).

**Table 1:** Physiological functions of SMXL proteins with the corresponding core signaling pathways and the manner (positive or negative), in which the phenotype is regulated by the SMXLs.

General process	Phenotype	Pathway	Regulation	Species	References
Seed germination	Seed germination	KAI2; MAX2; SMAX1	−	Arabidopsis	Shen et al., 2007; Waters et al., 2012; Stanga et al., 2013
Seedling establishment	Hypocotyl elongation	D14; MAX2; SMAX1/SMXL2	+	Arabidopsis	Waters et al., 2012; Wang et al., 2020b; Li et al., 2022
	Hypocotyl elongation	KAI2; MAX2; SMAX1/SMXL2	+	Arabidopsis	Shen et al., 2007; Sun and Ni, 2011; Waters et al., 2012; Stanga et al., 2013
	Mesocotyl elongation	D14; MAX2; SMXL6/7/8	+	Rice	Kameoka and Kyozuka, 2015; Zheng et al., 2020
	Mesocotyl elongation	KAI2; MAX2; SMAX1	+	Rice	Kameoka and Kyozuka, 2015; Choi et al., 2020a; Zheng et al., 2020
	Cotyledon expansion	KAI2; MAX2; SMAX1/SMXL2	+/−^a^	Arabidopsis	Sun and Ni, 2011; Stanga et al., 2013, 2016
	Cotyledon expansion	D14; MAX2; SMXL6/7/(8)	+/−^a^	Arabidopsis	Waters et al., 2012; Soundappan et al.,2015
Shoot development	Shoot branching	D14; MAX2; SMXL6/7/8	+	Arabidopsis	Gomez-Roldan et al., 2008; Umehara et al., 2008; Waters et al., 2012; Soundappan et al., 2015; Wang et al., 2015
	Shoot branching	D14; MAX2; SMXL7	+	Pea	Gomez-Roldan et al., 2008; de Saint Germain et al., 2016; Kerr et al., 2021
	Shoot branching	SMAX1	–^b^	Arabidopsis	Zheng et al., 2021
	Tillering	D14; MAX2; SMXL6/7/8	+	Rice	Umehara et al., 2008; Arite et al., 2009; Jiang et al., 2013; Zhou et al., 2013
	Tillering	SMXL6/7/8	+^c^		Liu et al., 2017
	Branch angle	D14; MAX2; SMXL6/7/8	−	Arabidopsis	Liang et al., 2016
	Shoot elongation	D14; MAX2; SMXL6/7/8	−	Arabidopsis	Soundappan et al., 2015; Liang et al., 2016
	Secondary growth	D14; MAX2; SMXL(6)/7/(8)	-^b^	Arabidopsis	Agusti et al., 2011; Bennett et al., 2016; Liang et al., 2016
Leaf development	Leaf length	D14; MAX2; SMXL6/7/8	−	Arabidopsis	Scaffidi et al., 2013; Soundappan et al., 2015
	Leaf length	KAI2; MAX2; SMAX1	+	Arabidopsis	Soundappan et al., 2015
	Petiole length	D14; MAX2; SMXL6/7/8	+	Arabidopsis	Scaffidi et al., 2013; Soundappan et al., 2015
	Leaf width	KAI2; MAX2; SMAX1	+	Arabidopsis	Soundappan et al., 2015
	Leaf senescence	D14; MAX2; SMXL6/7/(8)	−	Arabidopsis	Woo et al., 2001; Ueda and Kusaba, 2015; Bennett et al., 2016
Root development	Lateral root formation	MAX2; SMXL6/7/8	+	Arabidopsis	Kapulnik et al., 2011; Ruyter-Spira et al., 2011; Soundappan et al.,2015; Villaécija-Aguilar et al.,2019
	Lateral root formation	KAI2; MAX2; SMAX1/SMXL2	+	Arabidopsis	Villaécija-Aguilar et al., 2019
	Root skewing angle	KAI2; MAX2; SMAX1/SMXL2 and SMXL6/7/8	+^d^	Arabidopsis	Swarbreck et al., 2019; Villaécija-Aguilar et al., 2019
	Root straightness	KAI2; MAX2; SMAX1/SMXL2	−	Arabidopsis	Swarbreck et al., 2019; Villaécija-Aguilar et al.,2019
	Root diameter	KAI2; MAX2; SMAX1	−	Arabidopsis	Swarbreck et al., 2019; Villaécija-Aguilar et al.,2019
	Root hair formation and elongation	KAI2; MAX2; SMAX1/SMXL2	−	Arabidopsis	Villaécija-Aguilar et al., 2019
	Root hair elongation	KAI2; MAX2; SMAX1	−	Lotus	Carbonnel et al., 2020
	Primary root elongation	KAI2; MAX2; SMAX1	+	Lotus	Carbonnel et al., 2020
Drought tolerance	Stomatal closure	D14; MAX2; SMXL6/7/8	−	Arabidopsis	Bu et al., 2014; Van Ha et al., 2014; Lv et al., 2017; Kalliola et al., 2020; Yang et al., 2020a
	Anthocyanin/flavonoid production	D14; MAX2; SMXL6/7/8	−	Arabidopsis	Brewer et al., 2009; Ito et al., 2015; Walton et al., 2016; Li et al., 2020; Struk et al., 2021
	Anthocyanin/flavonoid production	KAI2; MAX2; SMAX1/SMXL2	−	Arabidopsis	Li et al., 2017; Bursch et al., 2021
	Cuticle formation	MAX2; SMXL6/7/8	−	Arabidopsis	Bu et al., 2014; Li et al., 2020
Osmotic stress tolerance	Osmotic stress tolerance	KAI2; MAX2; SMAX1/SMXL2	−	Arabidopsis	Li et al., 2022
	Osmotic stress tolerance	D14; MAX2; SMAX1/SMXL2	-^e^	Arabidopsis	Li et al., 2022
Symbiosis	AM fungi colonization	KAI2; MAX2; SMAX1	−	Rice	Yoshida et al., 2012; Gutjahr et al., 2015; Choi et al., 2020a

a *Indications that different SMXLs regulate this phenotype oppositely*.

b *Phenotype only found when SMXL is overexpressed*.

c *No mutant phenotype, only protein interaction data and effect on SPL expression*.

d *Involvement of SMXL6/7/8 not consistent*.

e *Unexplained opposite phenotype of smax1/smxl2 and smxl6/7/8 mutants*.

The KAI2-MAX2 signaling pathway has been proposed to only target SMAX1/SMXL2 for proteasomal degradation (Khosla et al., 2020; Wang et al., 2020b; Zheng et al., 2020). SMAX1 is directly involved in the regulation of seed germination (Stanga et al., 2013) and leaf development (Soundappan et al., 2015) and, together with SMXL2, in hypocotyl elongation (Stanga et al., 2013, 2016), lateral root density and root hair growth (Villaécija-Aguilar et al., 2019), and anthocyanin production (Figure 1; Table 1; Bursch et al., 2021). In lotus (*Lotus japonicus*), besides its role in KAI2-MAX2-SMAX1 signaling in root hair elongation, SMAX1 also seemingly regulates primary root length (Carbonnel et al., 2020). A new function for D14Like(OsKAI2)-D3(OsMAX2)-OsSMAX1 signaling was reported in rice, namely regulation of the arbuscular mycorrhizal fungi symbiosis establishment (Gutjahr et al., 2015; Choi et al., 2020a). Additionally, mesocotyl elongation in rice seedlings is also controlled by OsSMAX1, reminiscent of its influence on hypocotyl growth in *Arabidopsis* (Choi et al., 2020a; Zheng et al., 2020).

### SMXL3/4/5 Function Independently From KAI2 and D14 Signaling

The third and fourth SMXL clade, containing SMXL3 and SMXL4/5, respectively, in *Arabidopsis*, is the least studied, and its involvement in primary phloem formation was discovered relatively recently (Wallner et al., 2017; Wu et al., 2017). In addition, SMXL4/5, but not SMXL3, regulate secondary phloem development during radial growth, pointing to a possible functional distinction between the SMXL3 and SMXL4/5 clade (Shi et al., 2019; Wallner et al., 2020). SMXL4 also plays additional roles in gibberellic acid- and light-dependent regulation of flowering and seed setting, as well as in drought stress tolerance (Yang et al., 2015, 2016, 2020b). Contrary to other SMXL family members in *Arabidopsis*, SMXL3/4/5 are not involved in either KL or SL signaling and are not subjected to MAX2-dependent degradation (Wallner et al., 2017). Even though members of this clade are found throughout seed plants, their physiological roles in species other than *Arabidopsis* remain to be discovered (Walker et al., 2019).

### Functional Overlap Between SMXL Clades

Noteworthy, some phenotypes, such as leaf shape and lateral root density in *Arabidopsis*, and mesocotyl elongation in rice, are apparently under the control of both D14- and KAI2-dependent signaling, possibly to be interpreted as common outputs of the canonical D14-MAX2-SMXL6/7/8 and KAI2-MAX2-SMAX1/SMXL2 signaling complexes (Soundappan et al., 2015; Villaécija-Aguilar et al., 2019; Zheng et al., 2020). However, the attribution of a given SMXL subclade to either D14-MAX2 or KAI2-MAX2 partners might not be as clear-cut as initially thought. Indeed, the effect of KAI2 on root skewing depended on both SMAX1/SMXL2 and SMXL6/7/8, although these results were not consistent between different laboratories (Swarbreck et al., 2019; Villaécija-Aguilar et al., 2019). Also, D14-dependent inhibition of hypocotyl elongation was found to require SMAX1/SMXL2, rather than SMXL6/7/8 (Figure 1; Table 1; Wang et al., 2020b; Li et al., 2022). This finding is important, but must nonetheless be taken with caution, because this conclusion was based on the use of a synthetic SL analog (GR24^4DO^), and D14 signaling triggered by endogenous SLs is not involved in hypocotyl elongation (Nelson et al., 2011; Waters et al., 2012). However, recently, it was suggested that endogenous SLs might also employ D14-SMAX1/SMXL2 in another physiological context, namely the response to osmotic stress (Li et al., 2022).

Interestingly, in *Arabidopsis*, overaccumulation of SMAX1 could partly complement the increased shoot branching phenotype of *max2*, contrasting with the absence of a shoot branching phenotype in *smax1* (Zheng et al., 2021), but whether SMAX1 is involved in shoot branching regulation under physiological conditions remains to be seen. Along with the observation that AtSMAX1 is able to complement a *smxl45* double mutant, when expressed under the SMXL5 promoter, SMXL proteins from different subclades might possibly operate through a partially conserved mechanism/interaction network (Wallner et al., 2017).

Although knowledge on SMXL proteins is gradually increasing, several open questions on the activity and regulation of SMXLs still remain. The fact that SMXL3/4/5 are not subjected to MAX2-dependent degradation hints at the regulation of SMXL activity through another mechanism. Indeed, SMXL5 activity in sieve elements has been reported to be regulated at a translational level, through JULGI dependent formation of G-quadruplexes in *SMXL5* mRNA (Cho et al., 2018). However, it is not clear whether SMXL5 activity is controlled only in this manner and whether it is unique to SMXL3/4/5 or a general characteristic throughout the SMXL family. Additionally, novel insights on the physiological function of D14 and KAI2 signaling and their target SMXLs point toward an overlap and interaction between different SMXL clades, not yet recognized previously. Finally, the molecular mechanism by which SMXLs regulate physiological processes is still not completely uncovered. Through a compilation of recent findings on the phylogeny, activity, and regulation of SMXLs, we provide future cues to address the questions still surrounding these enigmatic proteins.

## Evolution and Phylogeny of SMXLs

**Figure 2:**
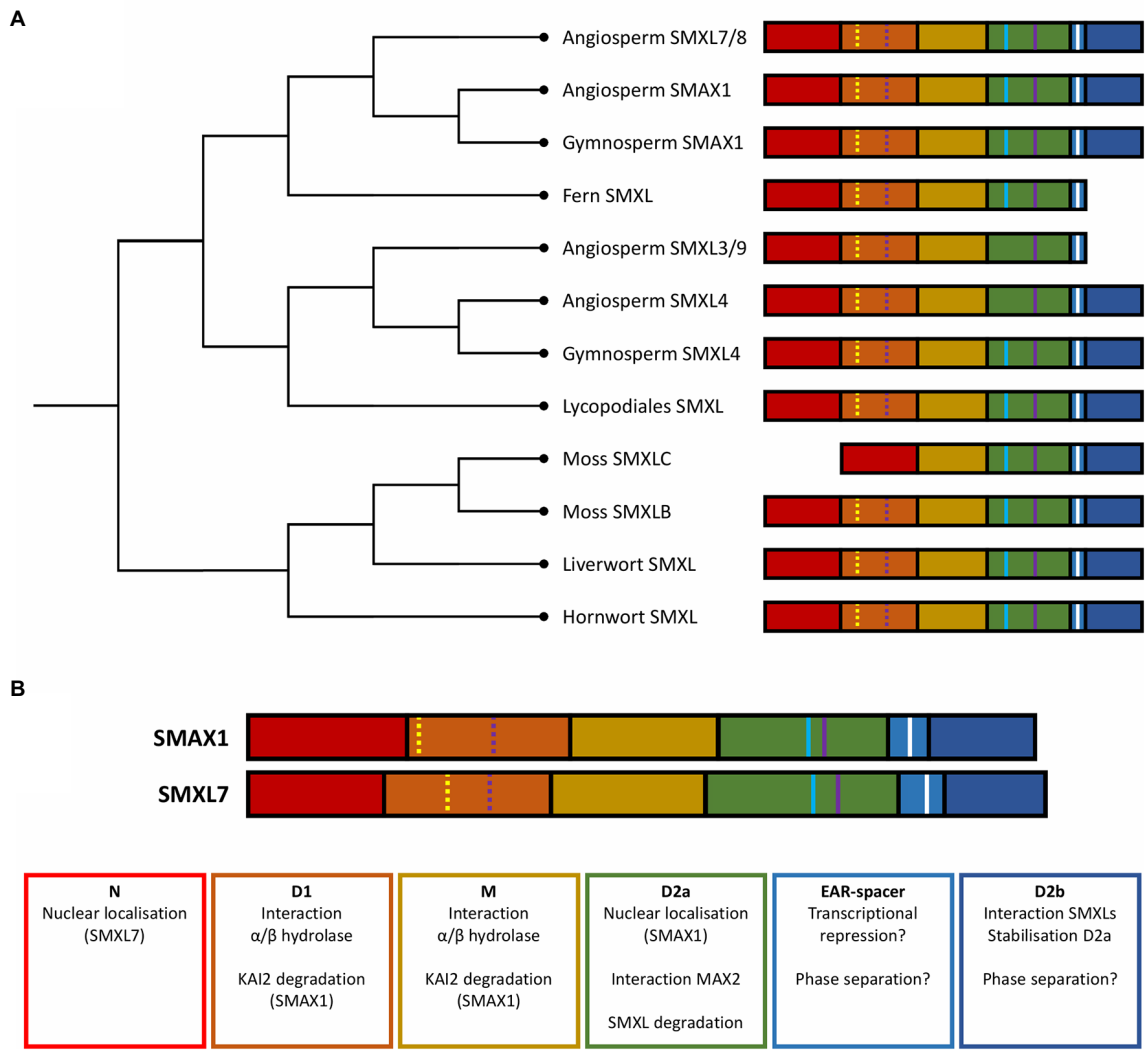
Evolution and structure of SMXL proteins. **(A)** Dendrogram showing the phylogenetic relationships between the major SMXL clades in different land plants. **(B)** Function of the structural domains of *Arabidopsis* SMAX1 and SMXL7. Colored blocks represent structural domains: N domain (red), D1 domain (orange), M domain (yellow), D2 domain NTPase 1 (D2a; green), a spacer containing the EAR-motif (light blue), and D2 domain NTPase 2 (D2b; dark blue). Colored lines represent short amino acid motifs: Walker A motif (yellow line), Walker B motif (purple line), EAR motif (white), and RGKT motif (blue).

Since the discovery of D53/SMXL proteins in rice (Jiang et al., 2013; Zhou et al., 2013; Zheng et al., 2020) and *Arabidopsis* (Stanga et al., 2013; Soundappan et al., 2015), SMXL family members have gradually been characterized in additional plant species, including wheat (Liu et al., 2017), apple (Li et al., 2018), woodland strawberry (Wu et al., 2019), lotus (Carbonnel et al., 2020), and pea (Kerr et al., 2021). Recent efforts to unravel the evolutionary history of this gene family have shown that SMXLs are both unique to and ubiquitously present in all land plants (Figure 2A; Walker et al., 2019). In angiosperms (a), SMXLs are grouped in four distinct clades, designated aSMAX1, aSMXL3/9, aSMXL4, and aSMXL7/8, which presumably arose from a single ancestral SMXL clade through two whole-genome duplication events, respectively at the origin of the seed plants and angiosperms (Moturu et al., 2018; Walker et al., 2019).

Based on genomic and *de novo* transcriptome assembly data from several species belonging to the bryophytes, lycophytes, and monilophytes, nonseed plants were concluded to generally possess only one ancestral SMXL clade, and most often a single *SMXL* copy (Walker et al., 2019). This ancestral SMXL is the most similar to the aSMAX1 clade and thought to be involved in the ancient KAI2-MAX2-dependent responses to KL (Bythell-Douglas et al., 2017; Walker et al., 2019). Recently, this hypothesis was supported by the discovery that in the liverwort *Marchantia polymorpha*, KAI2 and MAX2 homologs regulate thallus growth and gemma elongation through the degradation of the only SMXL homolog found in this species (Mizuno et al., 2021).

Despite the presence of the SL biosynthesis enzymes D27, CAROTENOID CLEAVAGE DIOXYGENASE (CCD) 7, CCD8, and MAX1 in most nonseed plants, the canonical SL receptor D14 is only found in seed plants (Bythell-Douglas et al., 2017; Walker et al., 2019), leading to the assumption that SLs first acted as symbiotic signals in the rhizosphere, rather than as plant development-regulating phytohormones (Kodama et al., 2021). Interestingly, most nonvascular land plants possess additional KAI2-like receptors, whereas in the moss *P. patens*, they appear to have evolved independently from D14 to act as SL receptors (Bythell-Douglas et al., 2017; Lopez-Obando et al., 2021). This SL sensitivity emergence in mosses is correlated with the acquisition of a second clade of SMXLs (Walker et al., 2019), allowing us to speculate that these additional SMXLs have been recruited as SL signaling targets (Figure 2A). Independently, a similar event has seemingly occurred at the origin of the angiosperms, when the SMAX1 lineage split into aSMAX1 and aSMXL7/8 (Walker et al., 2019). However, SL signaling in *P. patens* does not depend on MAX2, suggesting that this comparison is not entirely reliable and that functional examination of SMXL homologs in mosses is still needed to uncover their precise role (Lopez-Obando et al., 2021).

The currently designated canonical (i.e., D14- and MAX2-dependent) SL signaling has seemingly evolved at the source of the seed plants. Gymnosperms (g) only possess one gSMAX1 and one gSMXL4 clade, both originating from and very similar to the ancestral SMXL (Moturu et al., 2018; Walker et al., 2019). Based on data in angiosperms, the SMXL4 clade is not assumed to be involved in either KL or SL signaling, leading to the hypothesis that both pathways could target members of the gSMAX1 clade in gymnosperms (Wallner et al., 2017; Walker et al., 2019). This duplication of a single ancestral SMXL into a SMAX1 and SMXL4 correlates with the acquisition of two important traits, namely the formation of seeds and secondary growth (Linkies et al., 2010; Spicer and Groover, 2010; Walker et al., 2019). Especially interesting is that in *Arabidopsis* members of the SMAX1 clade are important for seed germination and seedling establishment, whereas the SMXL4 clade is involved in secondary phloem formation (Stanga et al., 2013; Shi et al., 2019; Wallner et al., 2020; Wang et al., 2020b). Hence, duplication and neofunctionalization of SMXLs might possibly have played a part in the development of these traits. Alternatively, because the main role of SMXL4 clade members apparently lies in vascular tissue formation (Wallner et al., 2017, 2020), we might consider that the SMXL4 clade possibly originated from subfunctionalization, rather than from neofunctionalization, and that the ancestral SMXL clade had already acquired a role in vascular development in Tracheophyta. To determine whether the SMXL4 clade or the other divisions of SMXLs in subclades originated through subfunctionalization or neofunctionalization, the recent, but still scarce, data on the SMXL phylogeny should be supplemented with functional insights into the roles of SMXLs in non-angiosperms.

A second whole-genome duplication at the origin of the angiosperms resulted in the further subdivision of the SMXL4 clade into aSMXL4 and aSMXL3/9. On the contrary, the SMAX1 clade diverged into aSMAX1, preserving its putative function in KL signaling, and aSMXL7/8, mainly functioning as targets for D14-dependent SL signaling. Further duplications of aSMXL7/8 and aSMXL3/9 in dicotyledonous plants resulted in SMXL7 and SMXL8, and SMXL3 and SMXL9, respectively. Finally, presumably at the origin of the Brassicaceae, SMXL2, SMXL6, and SMXL5 emerged from the dicot SMAX1, SMXL7, and SMXL4, respectively, together with the loss of SMXL9 leading to the SMXL diversity, as observed nowadays in *Arabidopsis* (Walker et al., 2019). Based on amino acid sequence identity, most of the divergence between different SMXL clades has been assumed to have happened during the evolution of angiosperms, possibly hinting at a need for neofunctionalization of these regulatory proteins (Walker et al., 2019).

## SMXL Are Plant-Specific Atypical Clp-ATPases

Early reports on SMXL proteins have highlighted that their domain organization and certain key motifs resembled that of members of the caseinolytic peptidase B (ClpB) ATPase family (Zhou et al., 2013). Clp proteins are present in all three domains of life: Bacteria, Archaea, and Eukaryotes. In bacteria, they are known to assemble in ATPase complexes that unfold proteins by using energy from ATP hydrolysis, functioning either as “proto-proteasomes” or chaperones in the removal of protein aggregates (Singh and Grover, 2010). Indeed, depending on the addition of an unrelated ClpP serine protease to the ATPase complex, unfolded proteins can subsequently be either degraded or refolded correctly (Kim et al., 2001).

Based on the presence of certain domains, Clp ATPase proteins can be divided in two distinct classes, but both classes contain a Clp-N domain at the N-terminus, apparently mainly involved in substrate recognition, often through the association with adaptor proteins, such as ClpS (Wojtyra et al., 2003; Mizuno et al., 2012; Zhang et al., 2012; Nishimura et al., 2013, 2015; Mishra and Grover, 2016). In class I Clp ATPases, the N-terminal domain is followed by two nucleotide-binding domains (NBDs), which are separated by a variable M domain. In contrast, class II Clp ATPases lack the M domain and contain only the C-terminal NBD2 (Kress et al., 2009). The NBDs are necessary for ATP hydrolysis and oligomerization in a hexameric pore complex and require two conserved motifs for their function, Walker A and Walker B (Gottesman et al., 1990; Schirmer et al., 1996). In addition, the NBD2 domain can also contain an IGF/L motif, the presence of which will grant the Clp ATPase the ability to interact with a ClpP protease, and thus to degrade the unfolded protein (Kim et al., 2001; Singh et al., 2001).

Besides SMXLs, plants possess three class I (ClpB, ClpC, and ClpD) and one class II (ClpX) Clp ATPase subtypes, as well as ClpP proteases and ClpS adaptors (Peltier et al., 2004). As previously shown for bacteria and yeast ClpB, plant ClpB proteins cannot interact with ClpP and are hence presumed to act exclusively as chaperones (Kim et al., 2001; Peltier et al., 2004). Indeed, in bacteria and eukaryotes, ClpB homologs are transcriptionally induced under heat shock conditions and they protect the cells against heat stress (Squires et al., 1991; Sanchez et al., 1992; Schirmer et al., 1994). Moreover, plant ClpB proteins can be cytosolic (Agarwal et al., 2002; Singh and Grover, 2010), whereas other Clp subtypes are generally localized to the chloroplasts or the mitochondria and contain a ClpP interaction motif (Nishimura and van Wijk, 2015). In these organelles, the ClpP complexes mainly perform a housekeeping function similar to that of the nuclear and cytoplasmic 26S proteasomes, i.e., the degradation of incorrectly neosynthesized proteins (Ali and Baek, 2020).

In SMXLs, the N-terminal domain containing a double Clp-N motif is globally conserved (Figure 2B; Jiang et al., 2013; Stanga et al., 2013; Zhou et al., 2013; Moturu et al., 2018; Walker et al., 2019). This is also true for NBD1 and NBD2 (D1 and D2), that also contain the Walker A and B motifs and are separated by an M domain (Soundappan et al., 2015; Moturu et al., 2018; Walker et al., 2019). The D2 domain, in turn, consists of two nucleoside-triphosphatase (NTPase) subdomains, with one most closely resembling the NBD of Clp ATPases. Characteristics that differentiate SMXL proteins from other Clp ATPases are the presence of an ETHYLENE-RESPONSE FACTOR Amphiphilic Repression (EAR) motif between the two NTPase subdomains in D2, as well as an elongated M domain (Jiang et al., 2013; Zhou et al., 2013; Soundappan et al., 2015).

SMXL proteins retain a domain organization and certain key motifs similar to ClpB proteins and also lack the IGF/L motif (Moturu et al., 2018). As such, SMXLs resemble more closely the ClpB ATPases, which act as chaperones rather than participating in proteolytic complexes, and, therefore, might potentially share the same molecular function. However, Clp ATPases have been shown to control a wide variety of processes, based on their diverging expression patterns and substrates (Frees et al., 2007; Nishimura and van Wijk, 2015). In general, chaperones can regulate transcription by influencing the late maturation steps of transcriptional regulators, effectively regulating their chromatin-binding ability (Morimoto, 2002; Cha et al., 2017; Roncarati and Scarlato, 2017; Gvozdenov et al., 2019). For instance, in rice, ClpB has been proposed to modulate gene expression through interaction with heat stress transcription factors (Singh et al., 2012). Similarly, SMXL proteins might be assumed to influence a transcriptional output through the stabilization of certain transcriptional regulators in an active conformation. Finally, like other Clp ATPases, SMXL proteins could function as hexameric chaperone complexes, as it was shown they can interact with each other (Liang et al., 2016; Khosla et al., 2020). The existence of such complexes has been suggested for SMXLs in rice, but further validation is still required (Ma et al., 2017).

At first sight, SMXLs seem to have diverged from their supposed ancestral role as ClpB chaperones. A possible chaperone activity for SMXLs has not yet been studied in detail, even though it could, for instance, account for the transcriptional regulation of target genes, as described above. As the molecular mechanism by which SMXLs function is still not completely resolved, research on the similarities and differences with Clp proteins might lead to new insights to address this question.

## SMXL Proteins are Composed of Structural and Functional Domains

As Clp proteins have been shown to be modular, with different structural domains responsible for diverse functional aspects of the proteins as a whole, it is interesting to examine the SMXL domains from the same perspective. Recently, different functional characteristics of AtSMAX1 have been attributed to certain parts of the protein (Figure 2B; Khosla et al., 2020). In short, the D1-M domain appears to be important for binding with D14 or KAI2 receptors, whereas the D2 domain is essential for KAR-induced degradation. In addition, the D2 domain can be divided into two functional subdomains that loosely correspond to the two NTPase domains discussed above: D2a, which mainly determines the nuclear localization of SMAX1, and D2b, which is seemingly involved in the interaction between SMXL proteins and in the stabilization of D2a (Khosla et al., 2020).

### The Function of a Conserved ClpN Domain Is Uncertain

Initially, the N domain of SMXLs was thought to enable nuclear localization, because nuclear localization signals (NLSs) are present in AtSMXL7 and OsSMAX1 (Liang et al., 2016; Choi et al., 2020a). Since SMXLs so far appear universally localized to the nucleus, this would fit the conservation of the N domain in these proteins (Zhou et al., 2013; Soundappan et al., 2015; Liang et al., 2016; Wallner et al., 2017; Khosla et al., 2020; Zheng et al., 2020; Mizuno et al., 2021). However, whereas the N domain has been demonstrated to be indeed responsible for the nuclear localization of AtSMXL7, the D2a domain seems to be necessary for the nuclear localization of AtSMAX1 (Liang et al., 2016; Khosla et al., 2020). Additionally, the N domain is broadly conserved among Clp ATPases as a whole, further hinting at additional roles, besides nuclear localization (Moturu et al., 2018).

### The D1 and M Domains Interact With the Receptors

In general, the D1, and even more so the M domains are less conserved among the SMXL clades and, in AtSMAX1 and AtSMXL7, they were shown to be critical for the binding with KAI2 and D14, respectively (Walker et al., 2019; Khosla et al., 2020). Possibly, the variation in the clade-specific D1-M region arose either from the required interaction with the respective receptor, or from the putative absence of interaction with either, as can be hypothesized for AtSMXL3/4/5 due to their independence from both SL and KAR signaling (Wallner et al., 2017). The presence of intact AtSMAX1 proteins is needed for the MAX2-dependent degradation of the isolated SMAX1_D2_ domain, suggesting that SMXL degradation only occurs when SMXLs can directly bind the KAI2 receptor *via* D1-M (Khosla et al., 2020). Additionally, the KAR-induced MAX2-independent degradation of KAI2 seems to require the presence of both SMAX1 and SMXL2, implying that the interaction between KAI2 and these SMXLs has an additional function in this process (Waters et al., 2015a; Khosla et al., 2020). A similar suggestion has been made after the discovery of a KAI2^D184N^ mutant (*kai2-10*) in *Arabidopsis*, which is unable to induce downstream signaling and hypersensitive to the aforementioned MAX2-independent degradation (Yao et al., 2018). As D184 lies next to the D14-MAX2-binding interface in AtD14, *kai2-10* might also be defective in its ability to interact with MAX2. Moreover, it cannot be excluded that this mutation affects the presumed interaction between KAI2 and SMAX1 or SMXL2, leading to rapid degradation of KAI2^D184N^ (Yao et al., 2018).

### The D2a Domain Regulates SMXL Stability and Interaction With MAX2

The involvement of the D2 domain in the MAX2-dependent degradation of SMXLs can be attributed to the presence of the Walker A motif. D2-Walker A is required in several species for the degradation of SMAX1 (Khosla et al., 2020; Wang et al., 2020b; Zheng et al., 2020; Mizuno et al., 2021) and SMXL6/7/8 (Jiang et al., 2013; Zhou et al., 2013; Soundappan et al., 2015; Wang et al., 2015; Liang et al., 2016; Struk et al., 2018; Kerr et al., 2021). Several publications term this motif P-loop or (F)RGKT, according to its structure or its amino-acid sequence, respectively (Zhou et al., 2013; Soundappan et al., 2015; Wang et al., 2015; Liang et al., 2016; Struk et al., 2018). Interestingly, for rice D53, affinity pull-down and size exclusion chromatography revealed that the D2 domain can interact with the D14 receptor, but only in a complex with D3 (OsMAX2) and with an intact RGKT motif (Shabek et al., 2018), implying that the D2 domain contains the interaction interface between D53 and D3 and that the RGKT motif is part of this interface.

Interestingly, the importance of the RGKT-dependent interaction between SMXL and MAX2 mainly lies in the stabilization of the ternary complex, whereas the main driving interactions of the signaling complex formation occur between activated D14 and the other signaling components. Indeed, for D53 and D3, a *rac*-GR24-independent interaction was only demonstrated by *in vitro* studies (Jiang et al., 2013; Wang et al., 2015). Moreover, no direct *rac*-GR24 dependent interaction was shown between MAX2 and SMXL7 through FRET-FLIM, as was demonstrated for D14-MAX2 and D14-SMXL7 (Liang et al., 2016).

Additionally, the RGKT motif was proposed to destabilize AtSMAX1 in a MAX2-independent manner, by conferring an inherent instability to the protein or by subjecting it to an additional degradation pathway (Khosla et al., 2020). Other SMXL members are probably also degraded in a MAX2-independent manner, but the biological significance remains tentative and challenging to elucidate. We can speculate that the MAX2-independent control of the level of SMXLs in plant cells might possibly trigger the sensitivity for their further degradation in response to MAX2-dependent signaling.

### SMXLs Act as Transcriptional Regulators Through an EAR Motif

Besides the RGKT motif, the D2 domain also contains the EAR motif that is conserved throughout all the SMXL clades (Moturu et al., 2018; Walker et al., 2019). The demonstrated purpose of this EAR motif is to enable interaction with proteins containing a C-terminal to Lissencephaly Homology (CTLH) domain (Szemenyei et al., 2008). In plants, CTLH domains are found in transcriptional corepressors, called TOPLESS (TPL)/TPL-Related (TPR), which associate with multiple transcription factors to regulate developmental processes, such as meristem maintenance, leaf growth and development, regulation of the circadian clock, seed germination, and stress response (reviewed in Plant et al., 2021). In hormone signaling pathways, at least for brassinosteroids, gibberellic acid, auxin, and jasmonate, recruitment of TPL/TPR has been shown to be a mechanism for repression of target genes (Szemenyei et al., 2008; Pauwels et al., 2010; Oh et al., 2014; Ryu et al., 2014; Fukazawa et al., 2015). TPL/TPR corepressors are proposed to inhibit gene expression through association with histone deacetylase proteins, which induce compaction of chromatin and gene silencing (Krogan et al., 2012; Wang et al., 2013; Ryu et al., 2014).

Interaction with TPL/TPR proteins has been confirmed for rice D53 and *Arabidopsis* SMAX1 and SMXL6/7/8 (Causier et al., 2012; Jiang et al., 2013; Soundappan et al., 2015; Wang et al., 2015; Struk et al., 2018). Additionally, transcriptional activity assays in *Arabidopsis* protoplasts revealed that SMXL6/7/8 were able to repress gene expression in an EAR-dependent manner (Wang et al., 2015). This observation sparked the first hypotheses on the molecular mechanism by which SMXLs might regulate downstream effects, namely repression of gene expression by interaction with a transcription factor and recruitment of TPL/TPR corepressors to the promoter region of target genes. Later research confirmed the role of SMXLs as transcriptional regulators by indicating that SMXL6 can repress transcription factors that control the expression of *BRANCHED 1* (*BRC1*), *TCP DOMAIN PROTEIN 1*, and *PRODUCTION OF ANTHOCYANIN PIGMENT 1* genes, regulating shoot branching, leaf shape, and anthocyanin production (Wang et al., 2020a). SQUAMOSA PROMOTER BINDING PROTEIN-LIKE (SPL) 9 and SPL15 were identified as the transcription factors interacting with SMXL6/7/8 in the regulation of *BRC1* expression, mirroring the interaction of D53 with IDEAL PLANT ARCHITECTURE 1 (IPA1), also an SPL transcription factor, in rice (Song et al., 2017; Xie et al., 2020). In turn, because IPA1 induces the expression of *TEOSINTE BRANCHED 1* (*TB1*; *OsBRC1*), this pathway has been assumed to be conserved between rice and *Arabidopsis* (Lu et al., 2013). However, whereas interaction with IPA1 is also necessary for D53 to repress its own transcription, presumably through TPL/TPR as a feedback mechanism, AtSMXL6 was shown to bind directly to the promoters of *AtSMXL6/7/8* (Song et al., 2017; Wang et al., 2020a). A key difference between members of the monocotyledonous and dicotyledonous D53/SMXL6/7/8 clade is the presence of two predicted, monocotyledonous-specific EAR motifs, of which one interacts with TPL and TPR proteins (Jiang et al., 2013; Zhou et al., 2013; Ma et al., 2017; Moturu et al., 2018). The function of this second monocot-specific EAR motif, or its relation to differences in the SMXL mechanism in monocots and dicots has not been uncovered yet.

Besides a clear role for SMXLs as transcriptional repressors, SMXLs have also been suggested to function through other mechanisms. Indeed, not all the SMXL6/7/8 responses require the presence of an intact EAR motif (Liang et al., 2016). Additionally, SL-dependent inhibition of shoot branching has been suggested to be partly regulated by the localization of PINFORMED (PIN) proteins to the plasma membrane (Shinohara et al., 2013; Liang et al., 2016). As the effect on the PIN localization is not sensitive to treatment with cycloheximide treatment, it had initially been proposed to be a non-transcriptional output (Shinohara et al., 2013). However, more recent results hint at a more indirect regulation of the PIN localization by D14 and KAI2 signaling, thereby not ruling out that the direct output of the pathways is transcriptional (Zhang et al., 2020; Hamon-Josse et al., 2022). As such, whether SMXLs also regulate the signaling output in a nontranscriptional manner is not entirely clear. Interesting perspectives could be provided by unraveling the way in which SMXLs regulate EAR-independent phenotypes, such as shoot angle, petiole, and leaf blade length (Liang et al., 2016) or conversely whether nontranscriptional output requires the EAR motif or not. SMXLs have also been proposed to possibly regulate the PIN localization through their EAR motif-driven interaction with other CTLH-containing proteins that are involved in endocytosis (Kobayashi et al., 2007; Tomaštíková et al., 2012; Waldie et al., 2014). Additionally, we could hypothesize that SMXLs might influence events outside of the nucleus by targeting proteins that shuttle between the nucleus and the cytosol. Further exploration of SMXL protein interaction networks might help to assess this assumption.

### The D2b Domain Confers Protein Stability and the Ability to Oligomerize

Finally, the C-terminal part of the D2 domain, termed as D2b, seemed important for the interaction between SMXLs in *Arabidopsis*, both for SMAX1, SMXL2, and SMXL7 (Khosla et al., 2020). SMXLs have been found to form homo-, heterodimers and possibly even hexamers (Liang et al., 2016; Ma et al., 2017). AtSMAX1 constructs containing D2a without D2b were apparently severely destabilized, even in the absence of exogenous treatment (Khosla et al., 2020). This observation implies that D2b-mediated oligomerization improves SMXL stability (Khosla et al., 2020).

### The Functional Implications of Absent Domains or Motifs

Different aspects of SMXLs can be loosely attributed to the different recognized structural domains. In general, most SMXLs possess the same structural domains that differ in degree of conservation between different clades (Walker et al., 2019). However, some exceptions provide unique opportunities to enhance our understanding of the function of these separate domains (Figure 2A). Members of the aSMXL3/9 and aSMXL4 clade, for instance, lost their RGKT-motif (Moturu et al., 2018; Walker et al., 2019). In *Arabidopsis*, SMXL3/4/5 are indeed not degraded by addition of *rac*-GR24 and the process they regulate is unaffected in the *max2* mutant, demonstrating they are neither targets of SL/KL signaling nor of MAX2-dependent degradation (Wallner et al., 2017). Although the absence of the RGKT-motif seemingly abolishes the interaction between SMXLs and MAX2, the interaction between SMXLs and their respective α/β-hydrolase can presumably still occur when the D1-M domain is present, as demonstrated for SMAX1^D1-M^ (Khosla et al., 2020). Noteworthy, α/β hydrolases belonging to the DLK23 clade, which are closely related to D14 and KAI2, are missing a canonical MAX2 interaction interface, and diverged from D14 at the origin of the seed plants, when also SMAX1 and SMXL4/5 diverged into separate clades (Bythell-Douglas et al., 2017; Végh et al., 2017; Walker et al., 2019). This lead to the hypothesis that the SMXL4 clade evolved as targets for these DLK23 receptors (Machin et al., 2020). In addition to the absence of the RGKT motif, members of the aSMXL3/9 clade also appear to have lost their C-terminal domain. Based on the role of SMAX1^D2b^, we can speculate that the loss of the D2b domain would render these SMXLs unable to oligomerize (Khosla et al., 2020). The D2b domain would also confer protein stability, but because of the RGKT motif absence, members of the aSMXL3/9 clade are presumably stabilized and protected from MAX2-independent degradation. In correlation with the missing D2b domain, AtSMXL3 is not completely functionally redundant to AtSMXL4 and AtSMXL5, and functions at different, though overlapping, stages in vascular development (Miyashima et al., 2019; Shi et al., 2019; Wallner et al., 2020).

Interestingly, SMXLs from ferns also lack a D2b domain but retain the RGKT motif (Walker et al., 2019). We could speculate that fern SMXLs are somehow stabilized, either through inherent, clade-specific features of the proteins, or by a difference in cellular context. Besides the presence of KAI2-MAX2 signaling components and SL biosynthesis genes, no information is available on the role and mechanism of SMXL proteins in ferns (Bythell-Douglas et al., 2017; Walker et al., 2019).

Finally, members of one of the SMXL clades in moss, dubbed SMXLC, lack the D1 domain (Walker et al., 2019), which might potentially correlate with a loss or change of their ability to interact with an α/β-hydrolase. Although KAI2-like receptors independently acquired SL sensitivity in *P. patens*, this SL perception and signaling occurs independently of PpMAX2 (Lopez-Obando et al., 2018, 2021). Although SMXLs would need their D1 domain in canonical SL signaling, the independently evolved SL signaling pathway in moss might also differ in this regard. Future research will teach us which specific role the different SMXLs play in KL and SL signaling pathways of these organisms and how the putative role of SMXLs in moss SL signaling differs from that in seed plants.

## SMXLs Might Be Regulated Through Their Ability to Enter Biomolecular Condensates

### SMXLs Participate in Subnuclear Condensates

Lately, the function and formation of cellular membraneless compartments has gained attention (Choi et al., 2020b). These compartments are commonly referred to as biomolecular condensates, because they represent a region of the nucleoplasm or cytosol, in which biomolecules, usually proteins and RNA, are spatially concentrated (Banani et al., 2017). Many types of condensates form through a physical process, called liquid–liquid phase separation (LLPS), in which a solution spontaneously demixes into two phases (Hyman et al., 2014; Choi et al., 2020b). Whether a protein can or will demix into a condensate is highly dependent on its properties and its concentration, as well as on the surrounding conditions, such as temperature and pH (Ruff et al., 2018). One of the general functions of condensate formation is to act as an integration point for environmental signals (Yoo et al., 2019). Additionally, these compartments sequester specific biomolecules, buffer biomolecule concentration, or concentrate components involved in a specific process (Cao et al., 2020; Pavlovic et al., 2020). Some condensates are commonly found in eukaryotic organisms, such as the nucleolus, nuclear speckles, Cajal bodies, and stress granules (Collier et al., 2006; Boisvert et al., 2007; Reddy et al., 2012; Cao et al., 2020). Plants additionally display specific condensates, including nuclear photobodies, AUXIN RESPONSE FACTOR 19/7 condensates in the cytosol of upper root cells, and condensates of FLOWERING LOCUS A in the nucleus (Van Buskirk et al., 2012; Fang et al., 2019; Powers et al., 2019).

Shortly after their discovery, some SMXL proteins were also found to be confined to distinct subnuclear condensates when transiently expressed in tobacco (*Nicotiana benthamiana*) leaves (Soundappan et al., 2015; Liang et al., 2016). To date, it is unclear whether this observation is an artifact due to protein tagging or overexpression, or also occurs in a physiological context. Either way, this aspect of SMXLs remains seriously understudied. Demonstration of a functional role for this subnuclear localization would open new interesting perspectives on the molecular mechanism by which SMXL proteins operate. Most importantly, it is still unknown whether SMXLs have the intrinsic ability to participate in LLPS, or whether SMXLs need to be localized in condensates to be functional, although their nuclear localization has been shown to be functionally relevant (Liang et al., 2016). Interestingly, AtSMAX1, AtSMXL7, and AtSMXL5 were shown to localize in nuclear condensates, suggesting that this characteristic is conserved across different SMXL clades, at least in angiosperms (Soundappan et al., 2015; Liang et al., 2016; Wallner et al., 2021). Whether this is true for all land plant species remains to be investigated, but the subnuclear localization of SMXLs might be an ancestral property. The remainder of this review will allude to speculative mechanisms by which SMXLs might form nuclear condensates and to some of possible functional implications.

### Multivalent SMXL-TPL/TPR Complexes Might Drive Phase Separation

**Figure 3:**
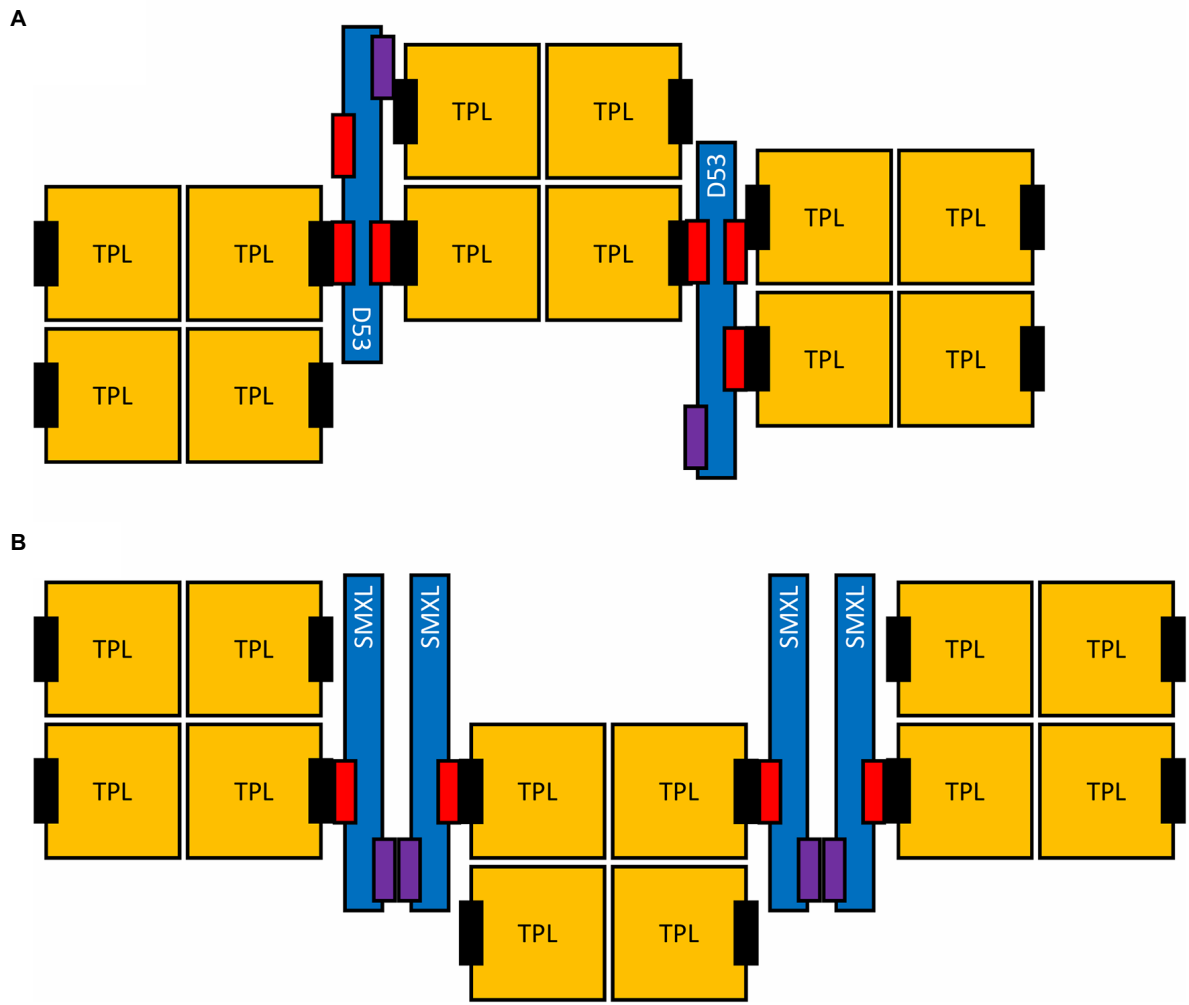
Possible higher-order assembly of SMXL proteins and TPL tetramers. Two alternative assemblies are shown, either specific for monocotyledous D53 **(A)** or for SMXLs in general **(B)**. The EAR motifs (red) and the putative oligomerization interface on SMXL proteins (purple), as well as the CTLH domain of TPL proteins (black) are indicated.

Interaction studies in tobacco leaves showed that AtSMXL7 is able to direct D14 to subnuclear condensates in a *rac*-GR24-dependent manner, implying this is where downstream SL signaling takes place (Liang et al., 2016). Additionally, the interaction of TPR2 with AtSMAX1 or AtSMXL7 also localizes to subnuclear condensates (Soundappan et al., 2015). Interestingly, a second EAR motif in rice D53 is able to simultaneously bind two TPR2 tetramers, of which each can interact with four EAR motifs (Ke et al., 2015; Ma et al., 2017). Multivalency is currently regarded as one of the main determining factors for biomolecules to phase-separate into condensates (Choi et al., 2020b). As D53 and TPR2 tetramers each have multiple interacting domains, they could potentially form multivalent units together, possibly forming higher-order aggregates (Figure 3A). Although the C-terminal EAR motif cannot bridge two TPR2 tetramers like the monocot-specific EAR motif, SMXLs have been proposed to be capable of interacting with each other as well and to form dimers or even hexamers (Ma et al., 2017; Khosla et al., 2020). Hypothetically, as such, the lack of a second EAR motif would be complemented, still providing the SMXL-TPR2 complex with multivalent interaction interfaces (Figure 3B). We hypothesize that the transcriptional control exerted by SMXL through association with TPL proteins might involve SMXL and TPL acting in a condensate. Such a mechanism is not unprecedented, because the transcription factor TERMINATING FLOWER (TMF) has recently been discovered to require redox-regulated reversible phase separation to repress gene expression as a so-called transcriptional condensate (Huang et al., 2021). If transcriptional repression by SMXL also depends on their ability to group in condensates, this could represent an additional level of SMXL activity control, besides their degradation. Moreover, as SMXL7 exerts EAR-dependent and EAR-independent functions (Liang et al., 2016), it is tempting to hypothesize that these functions correspond, respectively, to SMXL7 acting in a condensate or as “free” SMXL7. Indeed, it was noted that shoot phenotypes sensitive to SMXL7 overexpression depended on an intact EAR motif (Liang et al., 2016). According to our hypothesis, these phenotypes might be induced by SMXL7 entering condensates, which under the conditions tested might require higher SMXL7 levels than those in the wild type, as well as an EAR motif. SMXL levels lower than those in the wild type would not further inhibit their ability to form a condensate nor cause a phenotype. Conversely, phenotypes only associated with reduced SMXL6/7/8 levels could be complemented by SMXL7, regardless of the presence of the EAR motif and these phenotypes were not as strongly affected by SMXL7 overexpression (Liang et al., 2016). As a possible explanation, we might presume that once condensation starts, levels of “free” SMXL7 are buffered by condensation, because all excess SMXL7 would enter the condensate.

**Figure 4:**
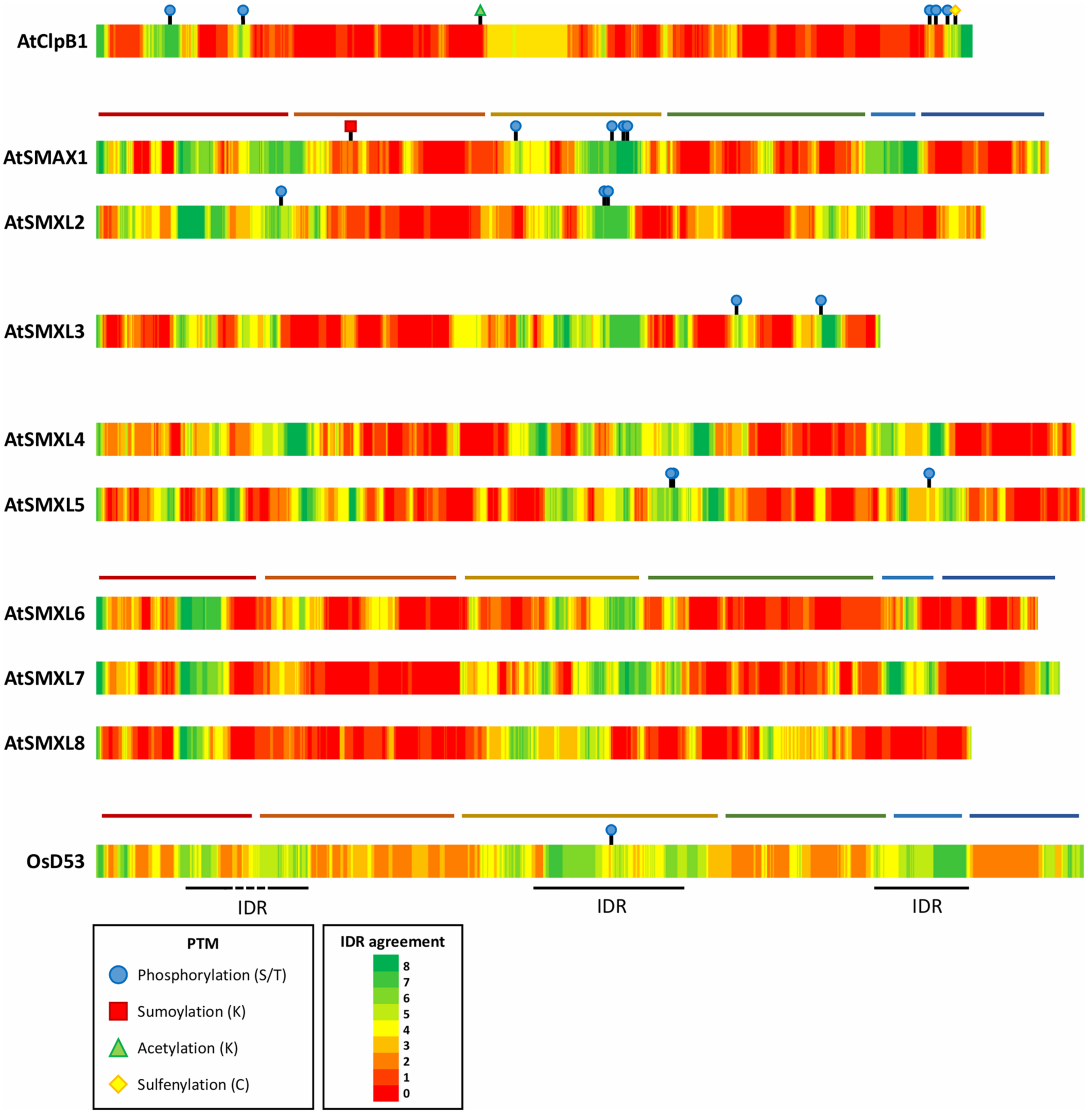
Posttranslational modifications and predicted disordered regions in *Arabidopsis* SMXLs, ClpB1, and rice D53. Predicted intrinsically disordered regions (IDRs) were acquired from different prediction tools collected by the D^2^P^2^ database (Oates et al., 2013). Per amino acid, how many prediction tools agree on the disorder are color indicated. When available, experimentally verified PTMs were obtained from the PTM viewer and displayed on the corresponding locations on the proteins (Willems et al., 2019). The domain structure of AtSMAX1, AtSMXL7 and OsD53 was added represented by horizontal, colored lines: N domain (red), D1 domain (orange), M domain (yellow), D2 domain NTPase 1 (D2a; green), a spacer containing the EAR-motif (light blue), and D2 domain NTPase 2 (D2b; dark blue).

Another property of SMXL proteins that could account for their presence in subnuclear condensates, is the occurrence of intrinsically disordered regions (IDRs). Intrinsically disordered proteins are characterized by their lack of a fixed three-dimensional structure and instead adopt a collection of different, dynamic conformations (Dunker et al., 2013). Noteworthy, protein disorder exists as a continuum and most proteins contain both folded domains and IDRs (Oates et al., 2013; van der Lee et al., 2014). Accordingly, IDR predictions extracted from the D^2^P^2^ database (discussed in Oates et al., 2013) reveal that in AtSMXLs and D53 the ordered D1, D2a, and D2b domains are generally separated by three IDRs corresponding to the M domain and a spacer between N and D1, and D2a and D2b, respectively, (Figure 4). Whereas protein disorder is not an absolute requirement for a protein to be part of a condensate, intrinsically disordered proteins are often driving LLPS (Posey et al., 2018; Martin and Holehouse, 2020). In proteins containing both ordered and disordered regions, the IDRs often confer the flexibility to a protein that is needed to engage in multiple dynamic interactions, using interaction interfaces that often reside in the folded domains (Choi et al., 2020b). Interestingly, the EAR motif is seemingly localized in the disordered spacer between D2a and D2b. As the EAR motif and the D2b domain are in close proximity, this flexible spacer might be essential to allow both interaction interfaces of SMXLs to aid in the formation of multivalent complexes with TPL/TPR.

### SMXL Condensates Could Act as Signaling Hubs

As biomolecular condensates often exist as a collection of hundreds of different biomolecules, SMXLs, D14, and TPL/TPR might not be the only components of the observed subnuclear condensates (Saitoh et al., 2004; Hubstenberger et al., 2017; Kosmacz et al., 2019). This implies that SMXLs could rely on other, possibly still unknown, interactors to enter condensates and are not necessarily the driving force behind the formation of the condensates. The scaffold and client hypothesis describes that biomolecules that are not essentially multivalent and not driving LLPS, i.e., the client, can be recruited to biomolecular condensates through an interaction with multivalent LLPS-driving scaffold molecules (Banani et al., 2016). Regardless of whether SMXLs direct LLPS through interaction with TPL or other biomolecules, we could speculate that the recruitment to or expulsion from the observed subnuclear condensates could act as an independent mechanism to modulate the SMXL activity in addition to proteasomal degradation. As addition of *rac*-GR24 does not seem to affect the SMXL7 localization into nuclear condensates (Liang et al., 2016), this added level of control might be employed by other signaling pathways, i.e., SMXLs might function as hubs for additional developmental environmental cues. SMXLs could thus perform a similar function as DELLA proteins, the primary repressors in the gibberellic acid signaling pathway, which had been shown to act as an integration point for almost all plant hormones (reviewed in Davière and Achard, 2016). Moreover, whereas the canonical gibberellic acid signaling pathway mainly regulates DELLA activity through proteasomal degradation, certain posttranslational modifications (PTMs), such as phosphorylation, sumoylation, and glycosylation, can also modulate DELLA functions, for example, in the drought stress response (reviewed in Blanco-Touriñán et al., 2020). For SMXLs, the functional importance of PTMs, other than ubiquitination, has not been investigated in detail, although proteomics experiments have revealed that several SMXLs in *Arabidopsis*, as well as rice D53, contain phosphorylated and sumoylated sites (Figure 4; Roitinger et al., 2015; Hou et al., 2017; Rytz et al., 2018; Mergner et al., 2020). Interestingly, the phosphorylation sites of SMXLs appear to be mainly localized in regions predicted to be IDRs, indicating that these IDRs, besides or instead of a potential role in LLPS, could also facilitate access to phosphorylation sites (van der Lee et al., 2014).

## Perspectives and Concluding Remarks

To start comprehending the understudied role of SMXLs in condensates and to test the proposed hypotheses, it is essential to first study the nature of these SMXL condensates. Most importantly, evidence that naturally expressed SMXLs enter subnuclear speckles in a physiological context is still missing. Importantly, whether SMXL-containing compartments overlap with known nuclear condensates is still unknown but could be investigated by means of colocalization with proteins known to localize to specific types of condensates. Additionally, assays have been developed to demonstrate whether a protein displays LLPS *in vitro*, which could help to detect whether SMXLs also drive LLPS, possibly in the presence of additional biomolecules or compounds, or under specific conditions. Additionally, interactomics experiments could help functionally to characterize SMXL condensates, hence uncovering possible interactions with proteins identified to localize to condensates and to drive LLPS. Finally, detailed localization studies could discover the specific circumstances in which SMXLs adopt this localization and the necessary protein domains or motifs. An interesting aspect of SMXLs to assess is their PTM landscape, both in relation to their subnuclear localization and their general function. Despite the identification of PTMs for some *Arabidopsis* SMXLs, very little is known on their impact on the SMXL function. This interesting, but understudied aspect of SMXLs, might very well provide the perspectives necessary to fill some holes in our knowledge of these puzzling proteins.

In conclusion, the rapid accumulation of insights on SMXL proteins opens a lot of interesting avenues to be studied. The characterization of functional domains in AtSMAX1 and AtSMXL7, as well as in SMXL homologs in other plant species that apparently lack one of these domains, could allow us to separately study the functional aspects of SMXLs that correspond to these distinct domains. Additionally, although functional insights on SMXLs in several angiosperm species are uncovered, they are still lacking in non-angiosperms, leaving an unexplored source of knowledge on these perplexing proteins. Research on the similarities and differences between SMXLs across land plants might ultimately help us to understand their array of physiological roles and molecular mechanisms.

Two understudied aspects of SMXLs remain their similarity to Clp ATPases and their localization to subnuclear condensates, which might be more relevant than has been appreciated thus far. As chaperones, SMXLs could regulate responses of the plant cell through the modulation of a wide array of target proteins, including transcriptional regulators. As members of biomolecular condensates, SMXLs could come in contact with multiple other factors, possibly functioning as integration points for more than one signaling pathway. Moreover, switching between the context of the nucleoplasm and a condensate might be an additional mechanism by which the SMXL function is regulated. The overview we provided might provide new avenues for the next steps in SMXL research.

## Author Contributions

AT was the main author of the manuscript. AG made significant contributions, especially to the second part of the review (SMXL Are Plant-Specific Atypical Clp-ATPases). AT, AG, SB, SG, and SS conceived and initiated the review and were involved in drafting and critically revising the manuscript. All authors contributed to the article and approved the submitted version.

## Funding

AT is indebted to the Research Foundation-Flanders for a predoctoral fellowship (3S015819). AG, SG, and SB acknowledge the support of Tournesol (Communauté Flandres-France) program N° 40660PF. The IJPB benefits from the support of Saclay Plant Sciences-SPS (ANR-17-EUR-0007).

## Conflict of Interest

The authors declare that the research was conducted in the absence of any commercial or financial relationships that could be construed as a potential conflict of interest.

## Publisher’s Note

All claims expressed in this article are solely those of the authors and do not necessarily represent those of their affiliated organizations, or those of the publisher, the editors and the reviewers. Any product that may be evaluated in this article, or claim that may be made by its manufacturer, is not guaranteed or endorsed by the publisher.
